# Structural basis for the acetylation of histone H3K9 and H3K27 mediated by the histone chaperone Vps75 in *Pneumocystis carinii*

**DOI:** 10.1038/s41392-019-0047-8

**Published:** 2019-05-10

**Authors:** Yiping Chen, Yang Zhang, Hui Ye, Yanshu Dou, Deren Lu, Xiaolu Li, Andrew H. Limper, Junhong Han, Dan Su

**Affiliations:** 1 State Key Laboratory of Biotherapy and Cancer Center, West China Hospital, Sichuan University, Chengdu, Sichuan P. R. China; 2 Center of Infectious Diseases, West China Hospital of Sichuan University, Chengdu, Sichuan P. R. China; 3 International Center for Translational Chinese Medicine, Sichuan Academy of Chinese Medicine Sciences, P.R. China, Chengdu, Sichuan P. R. China; 4 Thoracic Diseases Research Unit, Mayo Clinic College of Medicine, Rochester, MN USA

**Keywords:** Infectious diseases, Structural biology

## Abstract

Rtt109 is a histone acetyltransferase (HAT) that is a potential therapeutic target in conditioned pathogenic fungi *Pneumocystis carinii (P. carinii)*. The histone chaperone Vps75 can stimulate the Rtt109-dependent acetylation of several histone H3 lysines and preferentially acetylates H3K9 and H3K27 within canonical histone (H3–H4)_2_ tetramers. Vps75 shows two protein conformations assembled into dimeric and tetrameric forms, but the roles played by multimeric forms of Vps75 in Rtt109-mediated histone acetylation remain elusive. In *P. carinii*, we identified that Vps75 (PcVps75) dimers regulate H3K9 and H3K27 acetylation by directly interacting with histone (H3–H4)_2_ tetramers, rather than by forming a Vps75-Rtt109 complex. For PcVps75 tetramers, the major histone-binding surface is buried within a walnut-like structure in the absence of a histone cargo. Based on crystal structures of dimeric and tetrameric forms of PcVps75, as well as HAT assay data, we confirmed that residues 192E, 193D, 194E, 195E, and 196E and the disordered C-terminal tail (residues 224–250) of PcVps75 mediate interactions with histones and are important for the Rtt109 in *P. carinii* (PcRtt109)-mediated acetylation of H3K9 and H3K27, both in vitro and in yeast cells. Furthermore, expressing PcRtt109 alone or in combination with PcVps75 variants that cannot effectively bind histones could not fully restore cellular growth in the presence of genotoxic agents that block DNA replication owing to the absence of H3K9 and H3K27 acetylation. Together, these data indicate that the interaction between PcVps75 and histone (H3–H4)_2_ tetramers is a critical regulator of the Rtt109-mediated acetylation of H3K9 and H3K27.

## An unexpected interaction offers new hope for pneumonia

Structural biology insights into a chromosome-modifying enzyme could aid development of treatments for a potentially lethal form of fungal pneumonia. *Pneumocystis carinii* is a common cause of this disease in immune-compromised hospital patients, and researchers have focused on an enzyme called Rtt109 as a promising therapeutic target in this species. This enzyme regulates gene expression by chemically modifying chromosome-associated histone proteins, and researchers led by Dan Su and Junhong Han at Sichuan University have now dissected the mechanism underlying this process. They analyzed the structure of a “chaperone” protein, Vps75, which assists Rtt109-mediated histone modification. They learned that Vps75 enables this process by interacting with histones themselves, rather than partnering directly with Rtt109 as predicted. These findings suggest that clinicians could also potentially fight *P. carinii* infection with drugs that selectively target this Vps75-histone interaction.

## Introduction

Fungi are a growing cause of morbidity and mortality in the hospital-acquired infections.^1^ Pneumocystis pneumonia (PCP) is a severe cause of death in HIV and other immunosuppressed patients. *Pneumocystis carinii (P. carinii)* is the most common conditioned pathogenic fungus leading to PCP.^2^ Rtt109 in *P. carinii* (PcRtt109) is thought to be a potential therapeutic target for it absent in *Homo sapiens* and conserved in *Pneumocystis* species. Two histone chaperones, Vacuolar Protein Sorting 75 (Vps75) and the Anti-Silencing Function 1 protein (Asf1), are involved in the Rtt109-dependent histone H3K9, H3K27, and H3K56 acetylation. The site-specific mechanism for the stimulation of Rtt109 by Vps75 has remained unclear till now.

The Vps75 gene was originally identified in screening for genes involved in vacuolar protein sorting in *Saccharomyces cerevisiae* (*S. cerevisiae)*.^3^ Sequence alignment revealed that Vps75 is a member of the evolutionarily conserved nucleosome assembly protein 1/SE translocation (NAP1/SET) superfamily, members of which function as escorts for histone (H3–H4)_2_ tetramers and H2A–H2B dimers.^4^ Homologs of NAP1 have been identified in all eukaryotes, playing roles in the modulation of chromatin conformation, histone shuttling, histone modification, and nucleosome assembly. In yeast, NAP1 is originally identified as a histone chaperone and is primarily associated with histone H2A–H2B, thereby playing a pivotal role in chromatin assembly and disassembly.^5^ Recently, a new member of the NAP1/SET family, Ccp1 in *Schizosaccharomyces pombe* (SpCcp1), has been shown to cooperate with H2A.Z to prevent the ectopic formation of centromere-specific H3 variant chromatin.^6^ In humans, the NAP1/SET family consists of SET, Testis-specific Y-encoded protein 1 (TSPY), and TSPY-like (TSPYL) proteins, as well as several NAP1-like proteins.^7–9^ SET is one of the best-characterized NAP family members, forming a complex with TAF-Iα and pp32 to inhibit histone acetyltransferase (HAT) complexes and silence HAT-dependent transcription.^10^ TSPY binds cyclin-B, enhances the kinase activity of cyclin-B/CDK1 complexes,^11^ and is thought to mediate functions important for spermatogenesis.

Vps75 is a unique member of the NAP1 family in yeast, promoting the acetylation of histone H3 by the HAT Rtt109 (Regulator of Ty1 transposition protein 109).^4^ Initial studies in *S. cerevisiae* reported that Vps75 forms a complex with Rtt109 and that this complex acetylates histone H3 at H3K9, H3K14, H3K23, H3K27, and H3K56.^12,13^ Recent biochemical and structural studies have suggested that the site-specific efficiency of Rtt109 acetyltransferase activity is increased by binding to histone chaperones, namely, Vps75 and Asf1.^14,15^ Vps75 enhances histone H3 acetylation at H3K9 and H3K27, whereas Asf1 promotes H3K56 acetylation.^16^ The Vps75-Rtt109 complex is more stable than the Asf1-Rtt109 complex.^17^ Vps75-Rtt109 structures have been solved in which dimeric Vps75 interacts with one or two Rtt109 proteins (2:1 and 2:2 stoichiometric ratios).^18,19^ The Vps75 dimer enhances Rtt109 catalysis over 250-fold by stabilizing the catalytically active conformation.^20^ In the absence of Gcn5, the additional deletion of Rtt109 or Vps75 causes the loss of H3K9ac and H3K27ac and results in a growth defect.^21^ Therefore, Rtt109 must associate with the histone chaperone Vps75 to exhibit substrate specificity and enhance catalysis. However, the molecular mechanisms by which Vps75 dimers enhance Rtt109-mediated histone H3 acetylation remain elusive.

In addition to the dimeric form of Vps75, which associates with Rtt109, Vps75 also exists as a homotetramer in solution. Previous studies have shown that in the absence of a histone cargo, NAP1 and Vps75 form multiple oligomeric assemblies.^22^ A model of the Vps75 tetrameric structure has been proposed based on small-angle X-ray scattering data. In solution, two Vps75 dimers form a ring-shaped structure that conceals a highly acidic concave surface within the tetramer.^22^ In light of the observation that Vps75 and NAP1 form homotetramers in solution, Hammond, et al. co-crystallized Vps75 with Asf1-H3–H4 and revealed (at a resolution of 4 Å) a structure that contained two related symmetrical tetrameric forms of Vps75.^23^ However, it has been extremely difficult to obtain high-resolution crystal structure of the Vps75 tetramer.

In this study, we solved both dimeric and tetrameric structures of Vps75 from a pathogenic fungus, *P. carinii* (PcVps75), and identified a structural basis for the inability of PcVps75 to interact with PcRtt109. Two major histone-binding surfaces were identified for the PcVps75 dimer, including the disordered C-terminal tail and a highly negatively charged region that included residues E192, D193, E194, E195, E196, E203, and D204. In the PcVps75 tetramer, residues E192, D193, E194, and E196 were involved in a calcium-binding cluster that had essential roles in sustaining the PcVps75 tetramer by burying histone-binding surfaces within the structure. The site-directed mutagenesis of PcVps75 residues 192–196 to lysines abolished the interaction with histones and decreased the Rtt109-mediated acetylation of H3K9 and H3K27, both in vitro and in vivo. Therefore, we propose that Vps75 regulates the HAT activity of Rtt109 by affecting the interactions between histones and specific histone chaperones rather than by forming a complex with Rtt109. A model of how the histone chaperone, Vps75, promotes histone H3 acetylation by interacting with histone (H3–H4)_2_ tetramers is also presented. These results deepen our understanding of the mechanism by which a histone chaperone promotes the HAT activity of Rtt109, a potential therapeutic target in species of pathogenic fungi.

## Results

### Vps75 does not interact with Rtt109 in *P. carinii*

**Fig. 1:**
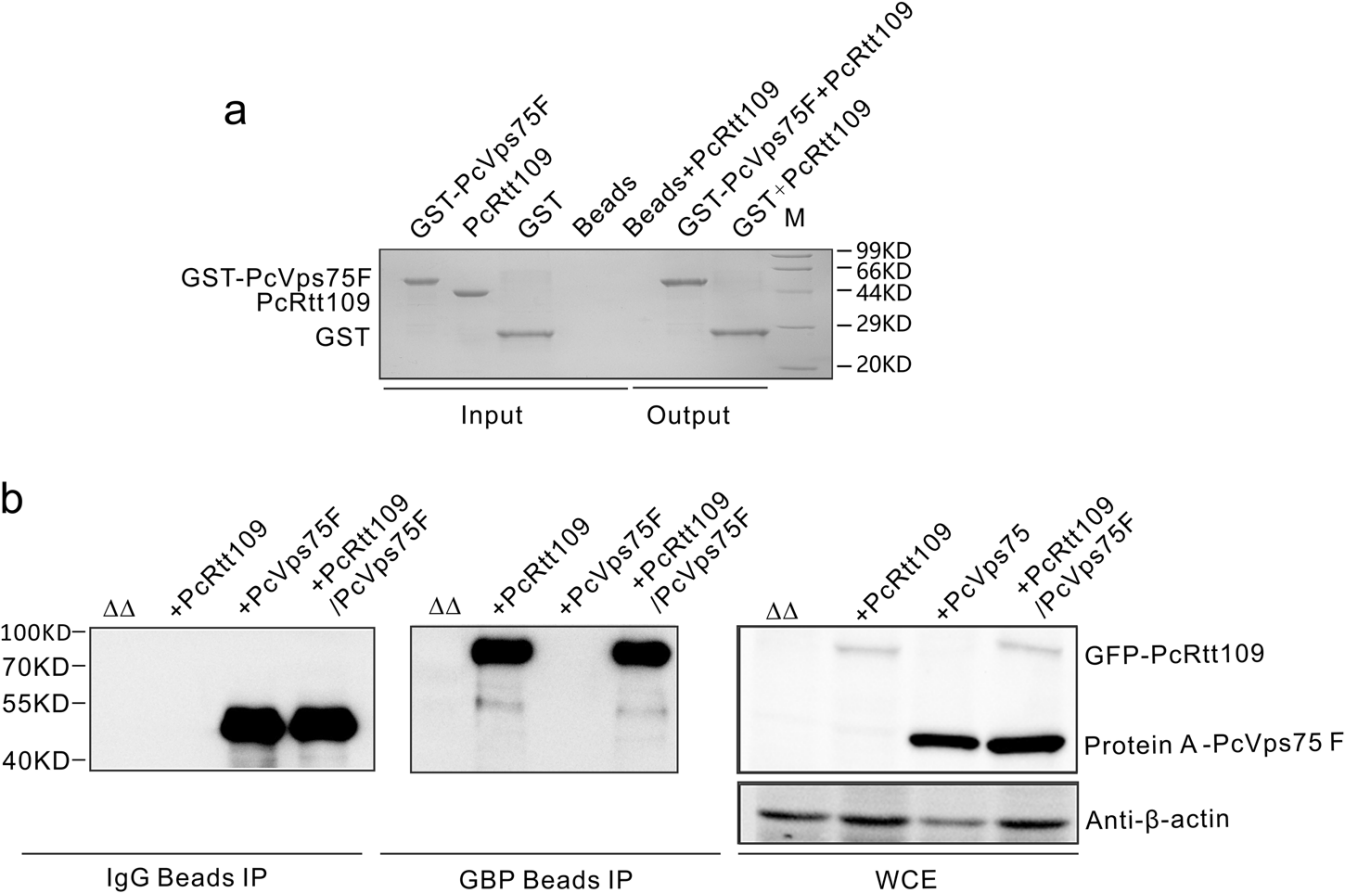
PcVps75 cannot form a complex with PcRtt109 in vitro or in vivo. **a** GST-PcVps75F, GST, and beads were incubated with excess amounts of PcRtt109. All proteins were detected by Coomassie staining. **b** GFP-Rtt109 and protein A-PcVps75F were expressed either together or separately in the double knockout yeast strain (*rtt109Δvps75Δ*) and detected with anti-GFP antibody. β-actin served as the loading control

Previous studies have reported that Vps75 can form a stable complex with Rtt109 in *S. cerevisiae* and that the Vps75-Rtt109 complex is required for the acetylation of several histone H3 lysine residues, including H3K9, H3K14, H3K23, H3K27, and H3K56.^15,24^ We initially attempted to purify the Vps75-Rtt109 complex from *P. carinii*. We labeled PcRtt109 (residues 1–375) and PcVps75F (residues 1–250, full length of PcVps75) the N terminus with 6-His and glutathione *S*-transferase (GST) tags, respectively, and co-expressed them in *Escherichia coli*. To our surprise, we failed to isolate the Vps75F-Rtt109 complex, even when using two independent affinity purification steps (Fig. 1a and S1a, b). Tag-free versions of these proteins were then prepared for analytical gel-filtration chromatography. Compared with the individual proteins, no peak shift was observed for the mixture (Fig. S1c, d), again indicating the lack of a complex. We also used isothermal titration calorimetry (ITC) to measure the interaction between PcVps75F and PcRtt109 and observed no changes in the thermodynamic parameters (Fig. S1e). These data all indicate that PcVps75F does not bind PcRtt109 in vitro.

We next asked whether PcVps75F interacts with PcRtt109 in vivo. We expressed PcVps75F and PcRtt109 in *S. cerevisiae*, as *P. carinii* cannot be grown in pure culture,^25^ and assessed the interaction between these proteins via co-immunoprecipitation assay. Specifically, Protein A-PcVps75F and GFP-PcRtt109 were expressed in the *rtt109*Δ *vps75*Δ strain of *S. cerevisiae*. Again, no stable complex was detected (Fig. 1b). The sequence alignment of Vps75 proteins from a number of fungi species revealed that PcVps75F shares low sequence identity (21–28%) with other Vps75 proteins (Fig. S2). To understand how this unique histone chaperone, PcVps75F, works with PcRtt109 to mediate HAT activity in the absence of a PcVps75F-PcRtt109 complex, we decided to characterize the structure of PcVps75F.

### Structure of the PcVps75 dimer and tetramer

**Fig. 2:**
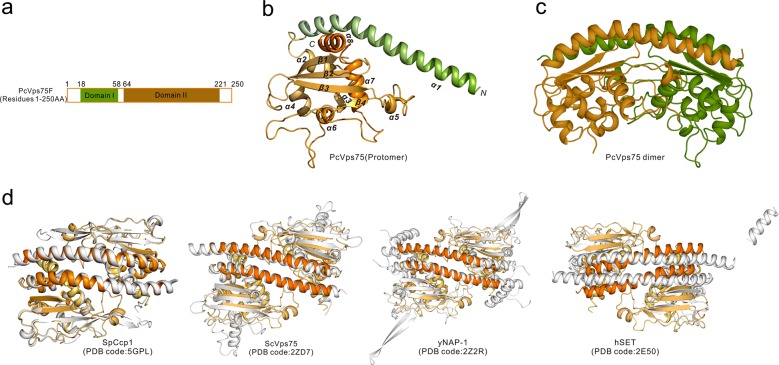
Overall structure of the PcVps75 dimer. **a** The domain organization of PcVps75F in *P. carinii*. Different colors identify unique regions or domains of PcVps75F. Domain I (green) and domain II (orange) appear in this structure. **b** Monomeric architecture of PcVps75∆C Elements of the secondary structure are labeled. **c** PcVps75∆C dimeric structure. Side view illustration of the PcVps75∆C dimer. **d** Superimposition of the PcVps75∆C dimer (orange) with SpCcp1, ScVps75, yNAP-1 and hSET separately (light gray)

The PcVps75 dimer adopts a classic “head-phone” topology, which is characteristic of the NAP family. The PcVps75 protomer contains two major structural domains, a long α-helix at the N terminus (domain I, residues 18–58, colored green) and a *α* + *β* globular domain (domain II, residues 64–221, colored orange) (Fig. 2a). An acidic disordered region (residues 224–250) at the C terminus is truncated owing to its flexibility. A search of the Dali server revealed that there are five structures associated with the NAP1 family that adopt a similar dimeric architecture in the PDB library,^26^ including SpCcp1 (PDB code: 5GPL), *S. cerevisiae* Vps75 (ScVps75) (PDB code: 2ZD7), yeast NAP1 (yNAP-1) (PDB code: 2Z2R), and *H. sapiens* SET (hSET) (PDB code: 2E50) (Fig. 2d and S3a–c). Comparing the structures of PcVps75 and ScVps75, domain II is more compact in PcVps75 than in ScVps75, as one of the α-helices in domain II of ScVps75 is substituted by a disordered loop (comprised of residues 161–175) in PcVps75 (Fig. S3a).

**Fig. 3:**
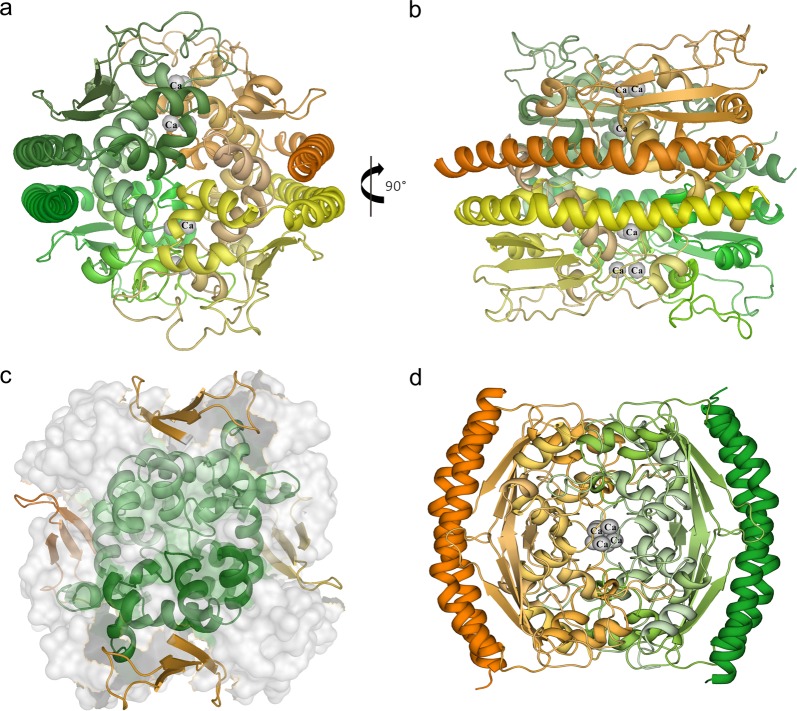
Overall structure of the PcVps75 tetramer. **a**, **b** PcVps75∆C-SeMet tetrameric structure. Chain a (orange), chain b (yellow), chain c (green), and chain d (dark green) are shown. **c** Interior core structure of the PcVps75 tetrameric structure. β1–β3 (orange) and α2–α4 and α6–α8 (green) are shown. The exterior of the core is shown as a surface and colored white. **d** Dimers I and II are colored orange and green. “Ca” represents a calcium ion in the center of the PcVps75 tetramer

The PcVps75 tetramer consists of two identical PcVps75 dimers (Fig. 3a, b), dimer I: A/B (orange and yellow) and dimer II: C/D (green and dark green). In such a way, the tetramer conceals the two large, negatively charged concave surfaces associated with the two PcVps75 dimers. Four quasi-equivalent protomers of PcVps75 are arranged in a solid, walnut-like structure. Two parallel α-helices (α_A_1/α_B_1, α_C_1/α_D_1) are present on each side of the PcVps75 tetrameric core, which is composed of four domain IIs. Within the core interior, each PcVps75 dimer contributes two domain IIs, including α-helices (α2–α8) and β–sheets (β1–β3), to form a golf ball-like structure. There are four anti-parallel β-sheets (β1–β3) located on the surface of the core. Inside of this core, four α-helix globular motifs are assembled by six α-helices (α2–α4 and α6–α8) with connecting loops from each motif arranged together (Fig. 3c). This generates a solid core inside the PcVps75 tetramer (Fig. 3d).

**Fig. 4:**
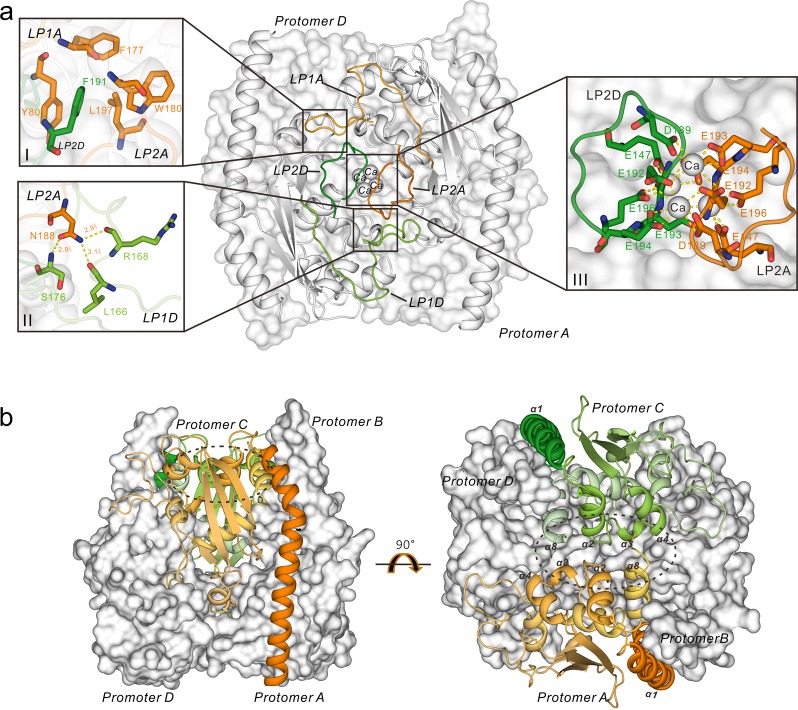
Interfaces within the PcVps75 tetramer. **a** Loop 1 and loop 2 in protomer D are colored green. Loop 1 and loop 2 in protomer A are colored orange. Loop 1 and loop 2 in protomer B are colored green. I: Detailed view of the hydrophobic interface between LP1A and LP2D. The key interacting residues in LP1A and LP2D are colored orange and green, respectively. II: The salt bonds between LP2A and LP1D are shown as dotted lines. The key interacting residues in LP2A and LP1D are indicated in orange and green, respectively. III: The electrovalent bonds between calcium and LP2A and LP2D are shown as dotted lines. The key interacting residues in LP2A and LP2D are indicated in orange and green, respectively. “Ca” represents calcium ions. **b** The second, minor interface between protomers B and C is circled with a dotted line. Protomer B is colored orange, and protomer C is colored green

The most noticeable difference between the PcVsp75 dimer and tetramer was that eight extended loops, namely, LP1A (residues 139–175 between α-helix α5 and α6 of PcVps75 protomer A), LP2A (residues 183–193 between helix α6 and α7 of PcVps75 protomer A), LP1B (residues 139–175 between α-helix α5 and α6 of PcVps75 protomer B), and LP2B (residues 183–193 between helix α6 and α7 of PcVps75 protomer B), stretch out from one PcVps75 dimer (PcVps75 dimer I) to form two major interfacing areas with similar loops (LP1D and LP2D) from PcVps75 protomer D and LP1C and LP2C from PcVps75 protomer C on the opposite PcVps75 dimer II. Among these, LP1A, LP2A, LP1D, and LP2D form one of two binding areas that maintain the tetrameric structure (Fig. 4a). In this area, residues Y80, F177, W180, and L197 on loop LP1A of protomer A form a hydrophobic interface that surrounds the highly conserved residue F191 on loop LP2D from the opposite PcVps75 protomer D (Fig. 4a–i). Meanwhile, the side chain of N188 on loop LP2A of protomer A forms a salt bond with the main chains of L166, R168, and S176 on loop LP1D of protomer D (Fig. 4a–II). Owing to the symmetric assembly, F191 on loop LP2A is surrounded by a similar hydrophobic interface formed by LP1D of protomer D, generating a narrow channel composed primarily of hydrophobic residues (Y80, F177, W180, and L197). In addition, the main chains of L166, R168, and S176 on loop LP1A form a salt bond with the side chain of N188 on LP2D. Above all, two long disordered loops (LP1A and LP1D) between α5 and α6 of PcVps75 protomers A and D surround LP2A and LP2D. This interface, which is composed of LP1A, LP2A, LP1D, and LP2D, is similar to the other interface between PcVps75 protomers B and C involving loops LP1B, LP2B, LP1C, and LP2C owing to the twofold axis of symmetry within the tetramer.

Two other minor interfaces of tetrameric PcVps75 are formed by globular motifs of PcVps75 protomers. One of these two surfaces associates with the globular motif of PcVps75 protomer A, including four α-helices (α2, α3, α4, and α8), and forms a hydrophobic surface. This surface is in contact with a similar region from the opposite PcVps75 protomer C (Fig. 4b). These two PcVps75 protomers come from PcVps75 dimer I and dimer II. Because of the twofold axis of symmetry that is perpendicular to the α-helix (α1) in tetrameric PcVps75, the other surface is similar to the first and is found between PcVps75 protomers B and D.

In the PcVps75 tetrameric structure, there are two large calcium-binding clusters composed of eight calcium ions and twenty-four acidic amino acids (147E_A/D_, 189D_A/D_, 192E_A/D_, 193D_A/D_, 194E_A/D_, and 196E_A/D_ from loops LP2A and LP2D; 147E_B/C_, 189D_B/C_, 192E_B/C_, 193D_B/C_, 194E_B/C_, and 196E_B/C_ from loops LP2B and LP2C) (Fig. 4a–III). The polyanion clusters binding to Ca^2+^ in the Vps75 tetramer neutralize the negative electric charge on the concave surfaces of the PcVps75 dimer, making it more convenient to cover two dimeric PcVps75s together in a tetrameric form.

### Structural basis of the interaction between Vps75 and Rtt109

Based on the structural analysis of two ScVps75–ScRtt109 complexes with different stoichiometries (PDB: 3Q35 and 3Q68), ScVsp75 contains three major contact surfaces that may contribute to these ScVps75–ScRtt109 complexes (surface 1, 2, and 3). However, only surfaces 1 and 2 are observed in both complexes (Fig. S4a, b). The sequence alignment of PcVps75 and ScVps75 revealed that only surface 2 is highly conserved in PcVps75 (Fig. S4c, d). Therefore, we reasoned that PcVps75F cannot bind PcRtt109 because PcVps75F has lost most major surfaces that contact PcRtt109. To our surprise, we found that PcVps75F could bind ScRtt109 (Fig. S5a). These data proved that surface 2 within PcVps75F is the most important region for interactions with ScRtt109.

It remained unclear why PcVps75F could not bind PcRtt109. We performed a multiple sequence alignment of Rtt109s from fungal species. The major sequence difference between PcRtt109 and ScRtt109 is a “loop-α-helix-loop” motif within ScRtt109 that is responsible for binding ScVps75 (Fig. S5e and S6). Therefore, we proposed that this motif mediates the ability of ScRtt109 to bind Vps75s, including ScVps75 and PcVps75F. To test this hypothesis, we constructed two truncated versions of ScRtt109: (1) ScRtt109ΔΗC, which lacks the “loop-α-helix-loop” motif (residues 123–170) and the C-terminal tail (resides 405–436), and (2) ScRtt109ΔC, which contains the “loop-α-helix-loop” motif but lacks the C-terminal tail. As expected, ScRtt109ΔHC did not bind PcVps75F, whereas ScRtt109ΔC did (Fig. S5b–d). Therefore, the “loop-α-helix-loop“ motif of ScRtt109 is a Vps75-binding motif. The motif in PcRtt109 is missing, so PcVps75F cannot form a complex with PcRtt109.

### The structural characterization of interactions between PcVps75 and histones

**Fig. 5:**
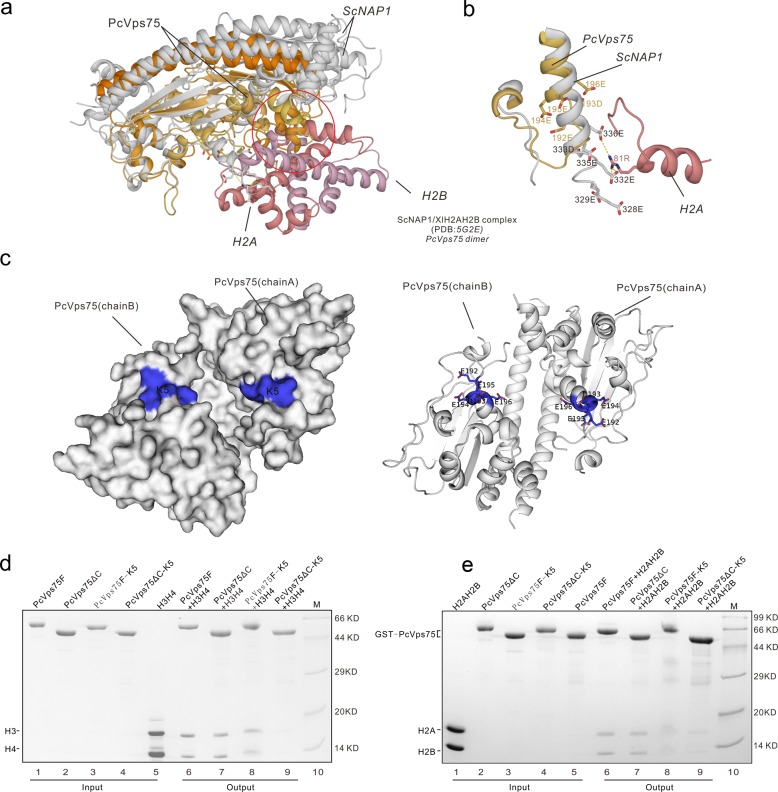
The major histone contact surface in PcVps75. **a**, **b** Superposition of PcVps75 with NAP1 in *S. cerevisiae* (ScNAP1) in the NAP1-H2A–H2B complex. PcVps75 is orange, ScNAP1 is silver, H2A is pink, and H2B is purple. The major interface between ScNAP1 and H2A is circled in red, and the homologous residues in PcVps75F within the interface are shown as sticks and colored orange. **c** The acidic region in α7 of PcVps75 is aligned with ScNAP1 and marked K5, shown as surface and sticks and colored blue. **d** GST-tagged PcVps75F, PcVps75F-K5, PcVps75ΔC, and PcVps75ΔC-K5 were incubated with excess (H3–H4)_2_ and stained with Coomassie Blue. **e** GST-tagged PcVps75F, PcVps75F-K5, PcVps75ΔC, and PcVps75ΔC-K5 were incubated with excess H2A–H2B and stained with Coomassie Blue

Vps75 binds tightly to both histone H2A–H2B and histone (H3–H4)_2_.^4^ A highly acidic concave surface between two globular domains of the Vps75 dimer is predicted to be the histone-binding surface owing to surface charge–charge interactions. However, a detailed description of how Vps75 interacts with histones, particularly from the structural perspective, remains elusive. Using the structure of the NAP1–H2A–H2B complex (PDB: 5G2E) and sequence alignment information, we predicted the location of a histone-binding surface within Vps75 (Fig. 5a–c). Within α helix α7 of PcVps75, a highly negatively charged region that includes E192, D193, E194, E195, E196, E203, and D204 may mediate interactions with histones. Similar to many histone chaperones, PcVps75 contains a long, disordered tail of acidic amino acids that interacts with basic histones, leading to charge neutralization.^27^ We therefore disrupted these two regions in a number of ways by constructing (1) a truncated version of PcVps75 that lacked this C-terminal acidic disordered region (CTAD, residues 224–250), which we called PcVps75∆C (residues 1–223); (2) a version in which acidic residues were changed to lysines (EDEEE192KKKKK), which we called PcVps75F-K5; and (3) a mosaic truncated mutant in which the CTAD is deleted from PcVps75F-K5, which we called PcVps75∆C-K5. No change in the secondary structural conformation of any of the truncated proteins was observed by circular dichroism spectroscopy (data not shown). PcVps75∆C and PcVps75F-K5 are partially impaired in their ability to interact with histone (H3–H4)_2_ and histone H2A–H2B compared with wild-type PcVps75F (Fig. 5d, e). However, the interaction between PcVps75∆C-K5 and histones is completely abolished. Finally, we revealed that helix α7, which is located within the highly acidic concave surface of PcVps75, and the CTAD domain are both necessary for PcVps75 to interact with histones.

### The Vps75-(H3–H4)_2_ complex promotes the Rtt109-mediated acetylation of histone H3K9 and H3K27

**Fig. 6:**
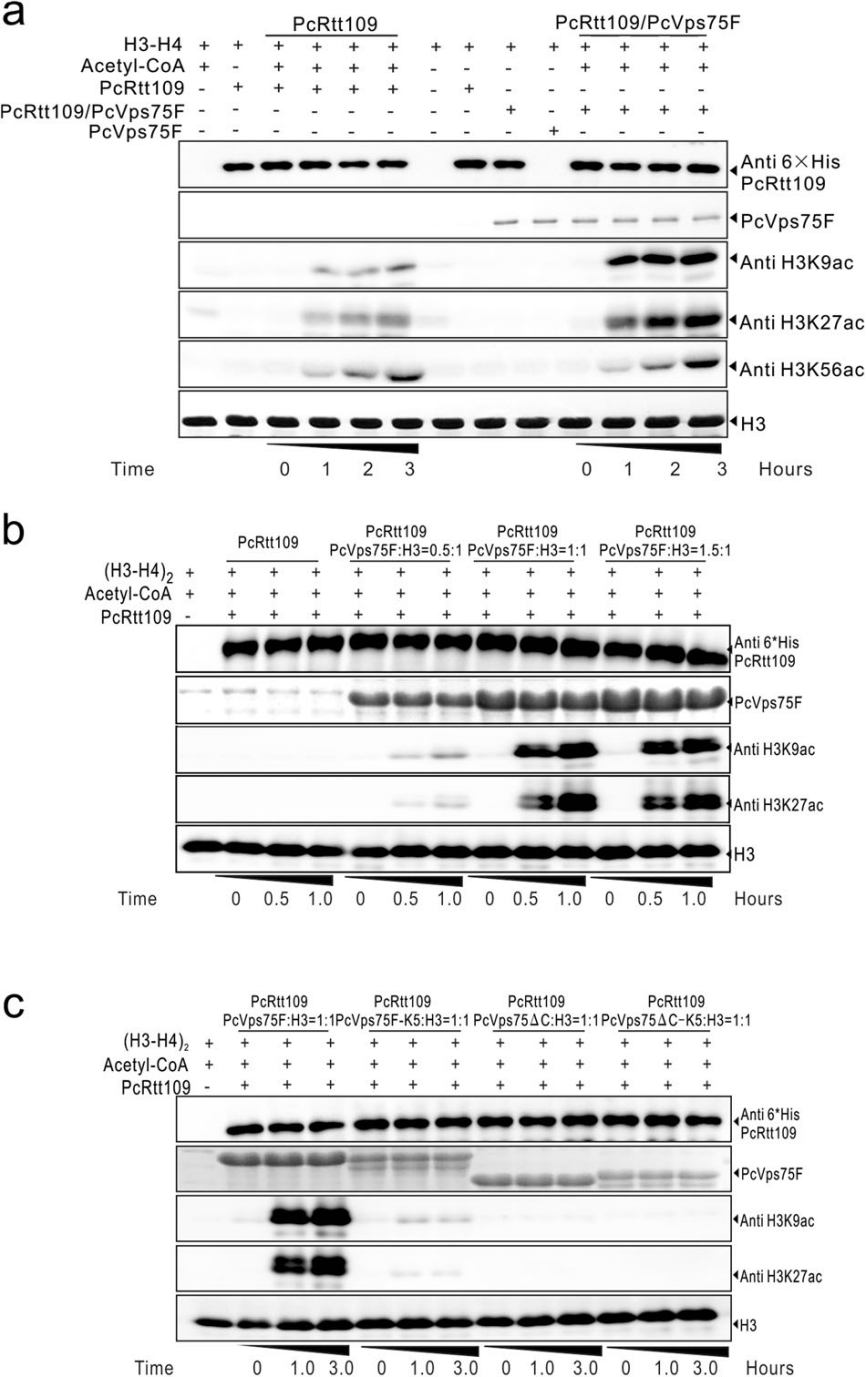
Vps75-dependent Rtt109 HAT activity on histone H3K9 and H3K27. **a** H3K9, H3K27, and H3K56 were acetylated by recombinant PcRtt109 and/or a mixture of PcRtt109 and PcVps75F, indicated as PcRtt109/PcVps75F. Acetyl-CoA was used in the HAT assay, and the presence of acetylated H3K9, H3K27, and H3K56 was determined by western blot analysis. The PcRtt109 proteins used in the assays were assessed by anti-His antibody (shown in the first panel). PcVps75F was assessed by Coomassie staining (second panel), while the substrate histone (H3–H4)_2_ was assessed by western blotting with anti-H3 antibody (last panel). **b**, **c** H3K9 and H3K27 were acetylated by recombinant PcRtt109 with **b** (H3–H4)_2_, PcVps75F:(H3–H4)_2_ (0.5:1), PcVps75F:(H3–H4)_2_ (1:1), or PcVps75F:(H3–H4)_2_ (1.5:1) and with **c** PcVps75F:(H3–H4)_2_ (1:1), PcVps75F-K5:(H3–H4)_2_ (1:1), PcVps75∆C:(H3–H4)_2_ (1:1), or PcVps75∆C-K5:(H3–H4)_2_ (1:1)

We used HAT assays to determine whether PcVps75 is necessary for PcRtt109 to acetylate H3K9, H3K27, and H3K56 in vitro. PcVps75F and PcRtt109 were used for these analyses. In our HAT assays, when PcRtt109 was incubated with PcVps75F, we detected higher levels of H3K9 and H3K27 acetylation via western blotting compared with PcRtt109 alone (Fig. 6a). H3K56 acetylation was unaffected in the absence of PcAsf1, in agreement with previous observations.^16^ By combining our HAT assays data and concerning protein interactions disability between PcVps75F and PcRtt109, we confirmed that the Vps75-Rtt109 complex was not required for PcRtt109-mediated HAT activity, particularly in the context of H3K9ac and H3K27ac. We further explored the possibility that a ternary complex of PcRtt109-(H3–H4)_2_-PcVps75F forms during histone acetylation, rendering the PcVps75F-PcRtt109 complex unnecessary. We mixed PcRtt109, PcVps75F, and (H3–H4)_2_ together and loaded them onto a gel-filtration column. Compared with the elution peaks of PcVps75F-(H3–H4)_2_, no further peak shifts were observed (Fig. S7). Therefore, PcVps75F-(H3–H4)_2_ cannot form a solid complex with PcRtt109, which is different from the Asf1-H3–H4-Rtt109 complex reported by Zhang L. et al.^28^ Together, these data indicate that the PcVps75F dimer, not the Vps75-Rtt109 complex, has a pivotal role in the Rtt109-mediated acetylation of H3K9 and H3K27.

To better characterize the ability of PcRtt109 to acetylate H3K9 and H3K27 in the presence of the PcVps75F-(H3–H4)_2_ complex, we varied the ratio of PcVps75F to histone (H3–H4)_2_ tetramers in combination with 6-His-tagged PcRtt109. PcVps75F dimers were first mixed with histone (H3–H4)_2_ tetramers at different molar ratios. The levels of H3K9ac and H3K27ac increased as the ratio of Vps75 to histone (H3–H4)_2_ increased (samples were incubated for 0, 1, 3, or 5 hours) (Fig. 6b). H3K9 and H3K27 were efficiently acetylated by PcRtt109 when the PcVps75F to (H3–H4)_2_ ratio was >1:1.

We also performed HAT assays with wild-type and mutant versions of PcVps75 to determine the extent to which the ability of PcVps75 to interact with histone (H3–H4)_2_ affected the activity of PcRtt109 to acetylate H3K9 and H3K27. PcVps75F, PcVps75-K5, PcVps75∆C, and PcVps75∆C-K5 were mixed with recombinant (H3–H4)_2_ tetramers at a molar ratio of 1:1. The PcVps75 mutants PcVps75∆C and PcVps75F-K5 do not promote the Rtt109-mediated acetylation of H3K9 and H3K27 compared with PcVps75F (Fig. 6c). The mosaic mutant, PcVps75∆C-K5, which could not interact with histone (H3–H4)_2_ tetramers, significantly decreased the activity of Rtt109 HAT towards H3K9 and H3K27. These data indicated that α helix 7 (α7) and the CTAD of Vps75 mediate binding to histone (H3–H4)_2_ and are both required to stimulate the Rtt109-mediated acetylation of histone H3K9 and H3K27 in vitro.

### Interaction between PcVps75 and histone H3–H4 promotes Rtt109 HAT activity in vivo

**Fig. 7:**
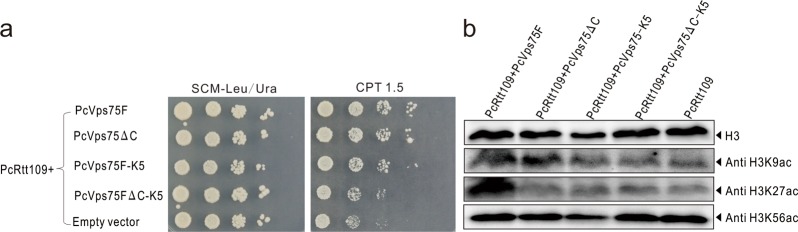
PcVps75 mutants affect the HAT activity of Rtt109 in vivo. **a** Tenfold serial dilutions of the indicated strains transformed with PcRtt109 or PcRtt109 with PcVps75-truncated plasmids were grown on SCM-Leu/Ura medium at 30 °C with 1.5 μm CPT. **b** Whole-cell lysates from *rtt109*Δ *vps75*Δ *gcn5*Δ transformed with different PcVps75 plasmids and PcRtt109 as indicated were analyzed via western blotting using antibodies against H3K9ac, H3K27ac, H3K56ac, and H3 (as loading control)

Previous studies have shown that Gcn5 and Rtt109 are both responsible for H3K9 acetylation in vivo.^29^ To study the role of the PcVps75-(H3–H4)_2_ interaction in regulating H3K9 and H3K27 acetylation and the cellular response to DNA damage, we used a triple deletion strain, *rtt109*Δ *vps75*Δ *gcn5*Δ*.* Cells were transfected with plasmids to express (1) PcRtt109, (2) PcRtt109 and PcVps75F, or (3) PcRtt109 and PcVps75 mutants. Whole-cell lysates were probed with antibodies against acetylated H3K9 and H3K27. In agreement with our previous results in vitro, H3K9ac and H3K27ac levels decreased in vivo when cells were transfected with plasmids to express (1) PcRtt109 and PcVps75-K5, (2) PcRtt109 and PcVps75ΔC, or (3) PcRtt109 and PcVps75ΔC-K5 compared with cells transfected with wild-type PcRtt109 and PcVps75F. As observed for the strain expressing PcRtt109 only, histones were not noticeably acetylated on H3K9 and H3K27 without PcVps75. As a control, the level of H3K56ac remained the same in all of these experiments (Fig. 7b). The fact that the PcVps75 mutants, both PcVps75-K5 and PcVps75ΔC-K5, are associated with reduced levels of H3K9ac and H3K27ac suggests that the interaction between PcVps75 and (H3–H4)_2_ is required for efficient acetylation of H3K9 and H3K27 by PcRtt109 in vivo. Moreover, genotoxic sensitivity assays showed that *rtt109*Δ *vps75*Δ *gcn5*Δ triple deletion strains that expressed PcVps75-K5 or PcVps75∆C-K5 in combination with PcRtt109 were more sensitive to DNA damage than cells expressing PcVps75F with PcRtt109 (Fig. 7a). This result indicates that the interaction between PcVps75 and histones plays the most important role in PcRtt109-mediated HAT activity and DNA damage repair.

## Discussion

Previous studies have revealed that the interaction between Vps75 and Rtt109 is required for Rtt109-mediated HAT activity because Vps75 maintains Rtt109 in a catalytically active form in *S. cerevisiae*.^19^ In this study, we confirmed that a long, disordered region within the main body of ScRtt109 (residues 123–170) is a Vps75-binding motif and is important for the formation of a Vps75-Rtt109 complex that involves ScVps75 or PcVps75. This disordered loop within native Rtt109 is converted to a “loop-α-helix-loop” conformation when interacting with Vps75. However, this motif is not conserved in all species of fungi, as approximately 60% of fungal Rtt109s lack this Vps75-binding motif (Fig. S8). This phenomenon is also discussed by Tang et al.,^19^ who speculate that Rtt109s that lack this motif interact with Vps75 through other unknown binding surfaces. Therefore, we assessed the interactions between PcVps75 and PcRtt109 in multiple ways and confirmed that PcVps75 cannot interact with PcRtt109, primarily because the Vps75-binding motif has been lost from PcRtt109.

After confirming that PcVps75 cannot form a stable complex with PcRtt109, we sought to determine whether PcVps75 regulates the Rtt109-mediated acetylation of histone H3K9 and H3K27, as Vps75 does in *S. cerevisiae*. Previous studies have indicated that Vps75 stimulates Rtt109 enzymatic activity and promotes histone acetylation at H3K9 and H3K27 by forming a complex with Rtt109.^15,30^ The site-specific acetylation of histone H3 by Rtt109 is dictated by the binding chaperone, in that Vps75-Rtt109 acetylates H3K9 and H3K27, whereas Asf1-Rtt109 acetylates H3K56.^24^ Our study showed that PcVps75 dimers can stimulate the HAT activity of PcRtt109 without forming a Vps75-Rtt109 complex, which displays a greater substrate specificity towards H3K9 and H3K27, two residues that are located in the N-terminal tail of histone H3. In contrast, H3K56ac is not dramatically affected by PcVps75 in the absence of Asf1. These results indicate that in *P. carinii*, the Rtt109-mediated histone acetylation of H3K9 and H3K27 is regulated by Vps75 dimers and that a Vps75-Rtt109 complex is not required.

**Fig. 8:**
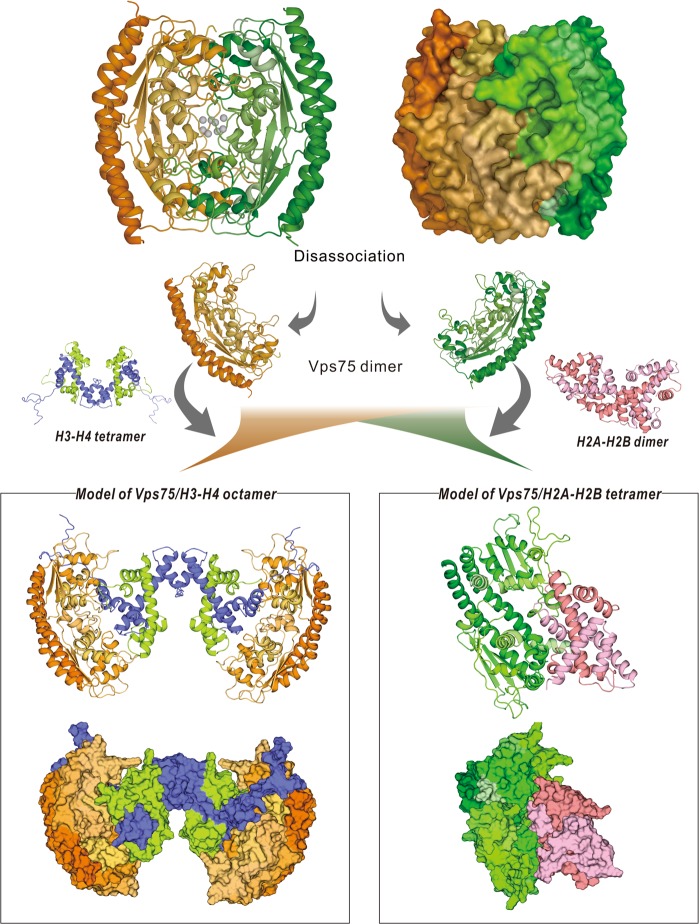
Model of PcVps75 interacting with histones. The models of PcVps75 interact with the (H3–H4)_2_ tetramer and H2A–H2B dimer

As PcVps75 mediates histone H3 acetylation without physically interacting with PcRtt109, we hypothesize that PcVps75 may influence histone H3 acetylation by binding (H3–H4)_2_ tetramers. It has been proposed that Vps75 interacts with histones to promote the HAT activity of Rtt109, but the mechanisms have remained elusive.^19,31^ In this study, two histone-binding surfaces were identified in PcVps75, including a negatively charged region located on the globular motif and the CTAD domain. The deletion of these two major histone-binding surfaces sharply reduced the ability of Rtt109 to acetylate histone H3K9 and H3K27, both in vitro and in the triple deletion strain (*rtt109*Δ *vps75*Δ *gcn5*Δ). Therefore, we showed that the interaction between PcVps75 and histones has a major role in regulating PcRtt109 HAT activity. We also discovered that PcVps75 increases the acetylation specificity towards H3K9ac and H3K27ac when the ratio between PcVps75 dimers and (H3–H4)_2_ tetramers is above 1:1. Considering this result and analytic gel-filtration data concerning the PcVps75-(H3–H4)_2_ complex, we propose a model in which two PcVps75 dimers interact with one (H3–H4)_2_ tetramer to form a hetero-octamer that is involved in histone acetylation at H3K9 and H3K27 (Fig. 8). The conformational changes to histone (H3–H4)_2_ induced by the interaction with PcVps75 may play a pivotal role in Rtt109-mediated histone acetylation. Of course, further characterization of the PcVps75-(H3–H4)_2_ structure is needed to confirm these hypotheses

The tetrameric structure of PcVps75 solved in this study revealed that histone-binding surfaces are obscured in tetrameric PcVps75. Several publications have previously described a ring-like model of the ScVps75 tetramer based on the dimeric structure of ScVps75. Bowman et al.^22^ used the small-angle X-ray scattering method to show that Vps75 and NAP1 adopt ring-shaped tetrameric conformations. Hammond et al.^23^ (2016) obtained crystals of the Vps75 tetramer by using Vps75-Asf1-H3–H4 co-crystallization and proposed two different forms of the Vps75 tetramer at 4Å resolution (PDB code: 5AGC). However, PDBePISA software analysis suggests that Vps75 only organizes into dimers in this structure (PDB code: 5AGC) (Table 1).^32^ In the tetrameric structure of PcVps75, two identical PcVps75 dimers form a solid, walnut-like structure rather than a ring-like structure. We were unable to compare the two different Vps75 tetramers due to the low resolution of the ScVps75 tetrameric model. However, it would not be surprising to see multiple forms of Vps75 tetramers owing to the low sequence identity between Vps75 homologies.

**Table 1:** Thermodynamics analysis of possible physiological assemblies of protein structure using the PDBePISA software.

	Composition	Surface area, sq. Å	Buried area, sq. Å	ΔG^INT^, kcal/mol	ΔG^diss^, kcal/mol
PcVps75	ABCD	35300	13550	− 93.2	40.4
	E_2_F_2_	35990	13930	− 91.0	38.8
ScVps75 PDB code: 5GAC	AB	25290	2820	− 29.0	17.1
	D2	24750	2690	− 25.7	15.7
	C2	24670	2400	− 22.6	11.4

The PcVps75 tetramer conceals the major histone-binding surface of PcVps75, which may be a way of preventing non-specific interactions. The histone-binding surface of PcVps75 is composed of a cluster of acidic residues, including 192E, 193D, 194E, 195E, and 196E. They are buried inside the PcVps75 tetramer, forming a large calcium-binding cluster (147E, 189D, 192E, 193D, 194E, and 196E) involving eight calcium ions (Fig. S9). We also noticed that the PcVps75 dimer is much more stable in a homogeneous state than tetrameric Vps75 in vitro. Therefore, we speculate that the tetrameric state may be affected by calcium ions within specific cellular environments. When tetrameric PcVps75 encounters histones, it can dissociate into two PcVps75 dimers, thereby exposing the histone-binding surfaces to the histone cargo. PcVps75 dimers could then interact with histone (H3–H4)_2_ tetramers or H2A–H2B dimers separately, performing pivotal roles in histone transportation in cells (Fig. 8). These models are also supported by recent studies, and it likely indicates that Vps75 interacts with histones to regulate histone modification.^5,33^

With non-specific therapeutic agents available currently, the development of resistance among infectious fungi, and the considerable adverse effects, the treatment of fungal infection, including Pneumocystis pneumonia, is challenging.^34,35^ Rtt109, as a histone acetyltransferase, is a potential therapeutic target in fungi, including Pneumocystis species. Several groups have tried to screen inhibitors based on Rtt109 but failed.^36,37^ Considering that PcVps75 can activate Rtt109 catalytic acetylating H3K9 and H3K27 and function in the DNA damage response by mediating the interaction with histones rather than forming a complex with Rtt109, new choices in drug design may be available based on the Rtt109-dependent histone acetylation pathway.

## Experimental procedures

### Plasmids and strains

cDNAs encoding PcVps75 and PcRtt109 were amplified by polymerase chain reaction (PCR) from the genomic DNA of *P. carinii* and cloned into the expression vectors pGEX 6p-1 and pET28a, respectively. ScRtt109 and ScVps75 from *S. cerevisiae*, (H3–H4)_2_ in the pET11a vector from *Drosophila melanogaster*, and H2A–H2B in the pRSF Duet1 vector from *H. sapiens* were obtained from Dr. Zhiguo Zhang. ScRtt109 and ScVps75 were cloned into pET Duet1 and pGEX 6p-1. All the mutations and truncations were prepared using the fast-cloning method,^38^ and primers encoding the mutated amino acids were verified by PCR and DNA sequencing (Sangon Biotech, China). For the in vivo binding assay, the following double deletion strains were used: W303A (*MATα leu2-3, 112 ura3-1, his3-11, 15, trp1-1, ade2-1, can1-100*); *rtt109*Δ *vps75*Δ (as W303A, but *vps75::kan; rtt109::kan*). Triple deletion strains *rtt109*Δ *vps75*Δ *gcn5*Δ (as W303A, but *rtt109::kan*; *vps75::kan; gcn5::natR*) were used for genotoxic sensitivity assays.

### Protein expression and purification

All expression plasmids were transformed into *E. coli* BL21(DE3) cells and grown at 37 °C in lysogeny broth supplemented with 25 mg/L ampicillin and 17 mg/L chloramphenicol. For expressing selenomethionine-labeled proteins, expression plasmids were transformed into the B834(DE3) strain (Novagen, German). Cells were grown in M9 medium supplemented with 20 amino acids and other nutrients.^39^ When OD_600_ levels reached 0.6–0.8, the cells were induced with a final concentration of 0.5 mm isopropyl *β*-d-1-thiogalactopyranoside and then cultured for an additional 18 hours at 16 °C with agitation (200 rpm). Cells were harvested and suspended in buffer containing 50 mm Tris, pH 8.0, 150 mm NaCl, and 1 mm ethylenediaminetetraacetic acid (EDTA) and then lysed, followed by affinity chromatography using GST-affinity columns (Glutathione-Sepharose beads, GE Healthcare, USA) and anion exchange chromatography with RESOURCE Q (anion exchange column, GE Healthcare, USA). Finally, the peaks of PcVps75 were collected and loaded onto a HiLoad Superdex 16/60 200 column (GE Healthcare, USA) equilibrated with 20 mm HEPES, pH 7.5, 150 mm NaCl, 1 mm EDTA, 1 mm DTT, and 5% glycerol (v/v). PcVps75 protein was concentrated to 25 mg/ml, frozen in liquid nitrogen and stored at − 80 °C for protein crystallization screening.

The purification procedure for ScVps75 was the same as for PcVps75 (described above). Because the full-length ScRtt109 showed extremely low expression in *E. coli*, the C-terminal tail (resides 405–436) was truncated to improve the protein yields. To purify PcRtt109, ScRtt109ΔC, and ScRtt109ΔHC, harvested cells were suspended in phosphate-buffered saline (PBS) containing 140 mm NaCl, 2.7 mm KCl, 10 mm Na_2_HPO_4_, 1.8 mm KH_2_PO_4_, pH 7.5, and 5% glycerol (v/v), followed by affinity chromatography with Ni-NTA affinity columns (Qiagen, German) and cation exchange chromatography with RESOURCE S (GE Healthcare, USA) and Superdex 200 (GE Healthcare, USA).

### Histone (H3–H4)_2_ tetramer purification

Purified *Drosophila* (H3–H4)_2_ tetramers were obtained as previously described.^40,41^ In brief, (H3–H4)_2_ in pET11a was transformed into *E. coli* strain BL21 (DE3) and induced with 0.4 mm for 1 hour at 37 °C. All subsequent operations were performed on ice. Following cell lysis, the pellets were suspended in 0.25 n HCl and homogenized. The resulting suspension was incubated at − 20 °C for 30 min, and the supernatant was dialyzed against Buffer A (50 mm HEPES, pH 7.0, containing 0.1 m NaCl, 1 mm EDTA, 1 mm DTT, and 10% glycerol (v/v)) and purified by cation exchange chromatography with Resource S and size exclusion chromatography on a Superdex 200 10/300 GL column (GE Healthcare, USA) with storage buffer (50 mm HEPES, pH 7.0, 500 mm NaCl, 1 mm EDTA, 1 mm DTT, and 10% glycerol (v/v)).

### Synthesis and purification of recombinant H2A–H2B dimers

Recombinant H2A–H2B in pRSF Duet1 was transformed into *E. coli* strain BL21 (DE3) and induced at an OD_600_ of 0.6–0.8 for 16 hours at 17 °C. The cells were centrifuged and resuspended in Buffer A (50 mm HEPES, pH 7.0 containing 500 mm NaCl, 1 mm EDTA, 1 mm DTT, and 10% glycerol (v/v)) and lysed. The lysate was filtered with a 0.45 µm filter membrane and loaded onto a cation exchange chromatography column (HiPrep SP 16/60, 20 ml, GE Healthcare, USA) followed by washing with 100 ml of buffer containing 0.6 m NaCl. H2A–H2B was eluted with buffer containing 1.4 m NaCl, and fractions containing H2A–H2B were pooled, centrifuged, and subjected to gel-filtration chromatography (Superdex 75 10/300 GL column, GE Healthcare, USA) with storage buffer (50 mm HEPES, pH 7.0, 500 mm NaCl, 1 mm EDTA, 1 mm DTT, and 10% glycerol (v/v)).

### Protein crystallization, data collection, and structure determination

A crystallization matrix (Hampton Research, Laguna Niguel, California, USA) was used for the initial screening of PcVps75F- and PcVps75-truncated proteins. An automatic drop dispensing and imaging robot (GRYPHON, Art Robbins instruments, USA) was used for the entire setup process. The sitting drop diffusion method was executed in autocrystallization. Truncated PcVps75ΔC (residues 1–223) generated a crystal with high diffraction quality in conditions from a Hampton Research SaltRx Kit (2.0 m ammonium citrate tribasic, 0.1 m BIS-TRIS propane, pH 7.0). For methionine is absent in PcVps75ΔC, two residues, E56 and N127, are mutated to methioine, generating PcVps75ΔCM. And the selenomethionine-labeled protein PcVps75ΔCM-SeMet crystals with suitable diffraction were harvested from conditions from the Hampton Research Crystal Screen (0.2 m calcium chloride dihydrate, 0.1 m HEPES sodium, pH 7.5, and 28% polyethylene glycol 400 (v/v)). Crystal optimization, including dimethyl sulfoxide (SIGMA, USA) and paraffin oil (Hampton Research, USA), was conducted after the initial crystal was found.

A set of SAD data on PcVps75ΔCM-SeMet was collected by using the BL17B beamline at The National Facility for Protein Science (NFPS) at Shanghai Synchrotron Radiation Facility, and the X-ray diffraction data for PcVps75ΔC were collected in Beijing Synchrotron Radiation Facility (BSRF).

Tetrameric PcVps75 crystals were obtained by growing selenomethionine-labeled proteins in conditions that included calcium chloride. The structure was determined using selenomethionine SAD phasing and then refined to final *R*_work_/*R*_free_ factors of 18.9%/24.2% at 2.1 Å resolution. Other parameters are included in Table 2. Using the tetramer structure of PcVps75 as the initial model, we solved the dimeric PcVps75 structure from a crystal of PcVps75ΔC by molecular replacement. The dimeric structure was refined to final *R*_work_/*R*_free_ factors of 18.9%/24.1% at 2.3 Å resolution with space group P4_1_. Other parameters are included in Table 3.

**Table 2:** PcVps75ΔCM-SeMet data collection and refinement statistics

PcVps75ΔCM-SeMet	
Data collection	
Wavelength (Å)	0.9785
Space group	*P2*_*1*_ *2*_*1*_ *2*
Unit-cell parameters (Å, °)	*a* *=* *141.7, b* *=* *76.4, c* *=* *130.5;* *α* *=* *β* *=* *γ* *=* 90.0
Resolution (Å)	50.00–2.09 (2.13–2.09)
*R*_merge_^a^ (%)	8.4 (56.7)
Average *I/σ(I)*	23.8 (3.8)
No. of observed reflections	1.111,487 (1147)
No. of unique reflections	159,739 (56,203)
Completeness (%)	100 (100)
Redundancy	7.0 (7.0)
Matthews coefficient (Å^3^ Da^−1^)	2.54
Solvent content (%)	51.52
Molecules per asymmetric unit	6
Refinement	
Resolution (Å)	48.26–2.09
*R*_work_/*R*_free_	0.19/0.24
Ramachandran favored (%)	95.87
Ramachandran allowed (%)	4.13
Ramachandran outliers (%)	0
No. of atoms	
Protein	10342
Ligand/ion	43/12
Water	232
Wilson B value	37.82
R.m.s. deviations	
Bond lengths (Å)	0.008
Bond angles (°)	1.085

^a^*R*_merge_= $\mathop {\sum}\nolimits_{hkl} {\mathop {\sum}\nolimits_i {\left| {I_i\left( {hkl} \right) - \left\langle {I\left( {hkl} \right)} \right\rangle } \right|/\mathop {\sum}\nolimits_{hkl} {\mathop {\sum}\nolimits_i {I_i(hkl)} } } }$, where *I*_*i*_ (*hkl*) is an individual intensity measurement, and $\left\langle {I\left( {hkl} \right)} \right\rangle$ is the average intensity for all *i* reflections

**Table 3:** PcVps75ΔC data collection and refinement statistics

	PcVps75ΔC
Data collection	
Wavelength (Å)	1.000
Space group	*P*4_1_
Unit-cell parameters (Å, °)	*a* *=* *b* *=* *66.4, c* *=* *99.4;* *α* *=* *β* *=* *γ* *=* *90.0*
Resolution (Å)	50.00–2.30 (2.35–2.30)
*R*_merge_^a^ (%)	5.0 (37.2)
Average *I/σ(I)*	37.1 (4.5)
No. of observed reflections	1,47,431 (8932)
No. of unique reflections	19,234 (1276)
Completeness (%)	99.8 (100)
Redundancy	7.7 (7.6)
Matthews coefficient (Å^3^ Da^−1^)	1.88
Solvent content (%)	34.49
Molecules per asymmetric unit	2
Refinement	
Resolution (Å)	46.96–2.30
*R*_work_/*R*_free_	0.18/0.24
Ramachandran favored (%)	96.24
Ramachandran allowed (%)	3.49
Ramachandran outliers (%)	0.27
No. of atoms	
Protein	3173
Ligand/ion	3/0
Water	94
Wilson B value	36.09
R.m.s. deviations	
Bond lengths (Å)	0.009
Bond angles (°)	1.201

^a^*R*_merge_ =$\mathop {\sum}\nolimits_{hkl} {\mathop {\sum}\nolimits_i {\left| {I_i\left( {hkl} \right) - \left\langle {I\left( {hkl} \right)} \right\rangle } \right|/\mathop {\sum}\nolimits_{hkl} {\mathop {\sum}\nolimits_i {I_i(hkl)} } } }$, where *I*_*i*_ (*hkl*) is an individual intensity measurement, and $\left\langle {I\left( {hkl} \right)} \right\rangle$ is the average intensity for all *i* reflections

### Protein–protein interaction: GST pulldown and ITC experiments

GST-tagged PcVps75F and ScVps75, 6-His-tagged PcRtt109, ScRtt109ΔC, ScRtt109ΔHC, and histones (H3–H4)_2_ and H2A–H2B were expressed in *E. coli* BL21(DE3) and purified separately, as described above. A gel-filtration column was used to improve the purity of recombinant GST-tagged PcVps75F, ScVps75, 6-His-tagged PcRtt109, ScRtt109ΔC, ScRtt109ΔHC, (H3–H4)_2_ tetramer, and H2A–H2B dimer to over 95%. GST-tagged PcVps75 and ScVps75 were incubated with GST-affinity beads on ice for 30 min in binding buffer (50 mm Tris, pH 8.0, 500 mm NaCl, 1 mm EDTA and 1 mm DTT). For the interaction assay between PcVps75 and PcRtt109, the salt concentration in binding buffer varied from 10 mm to 500 mm. The excess protein was thoroughly washed away with binding buffer. A total of 10 µl of 5 mg/ml Rtt109, ScRtt109ΔC, ScRtt109ΔHC, or histones was mixed with the beads with GST-tagged PcVps75 and ScVps75, respectively. After a 1-hour incubation on ice, the beads were washed thoroughly and then analyzed using sodium dodecyl sulfate polyacrylamide gel electrophoresis (SDS-PAGE).

ITC experiments were performed at 16 °C on an iTC200 calorimeter from MicroCal™ (Malvern, British). The concentration of each protein sample was determined from the respective absorbance at 280 nm (Nanodrop, Thermo, USA). Then, 400 μm PcRtt109 was titrated into a sample cell containing 40 μm of PcVps75.

### Analytic gel filtration

Analytic gel filtration was performed to investigate the interaction between Vps75, (H3–H4)_2_, H2A–H2B, and Rtt109 using a Superdex 200 10/300 GL column (GE Healthcare, USA) equilibrated with PBS (140 mm NaCl, 2.7 mm KCl, 10 mm Na_2_HPO_4_, and 1.8 mm KH_2_PO_4_, pH 7.5), and the whole process was conducted at 4 °C. Protein samples were at a concentration of ~ 10 mg/ml. A series of proteins with accurate molecular weights (Gel Filtration Standard, BioRad, USA) were used as the standards in the molecular weight calculation. The molecular weight was calculated using the following equation:

$$V_{\mathbf{e}}{\bf{ = - }}b^\prime {\mathbf{lg}}M{\mathbf{r}} + c^\prime$$

*V*e*: the volume at which the intermediate molecules elute; M*r*: molecular weight*.

### In vitro HAT assays

HAT assays were performed using recombinant (H3–H4)_2_ tetramers, acetyl-CoA (SIGMA, USA), and PcRtt109, PcVps75F, and PcVps75 truncated proteins in vitro. Samples were incubated at 25 °C for different times in 50 µl of reaction mixture containing 50 mm HEPES, pH 7.0, 1 mm EDTA, 1 mm DTT, 5% glycerol (v/v) and 200 µm acetyl-CoA. When indicated, recombinant (H3–H4)_2_ (20 µm), PcVps75F/(H3–H4)_2_ (molar ratio at 0.5:1, 1:1, or 1.5:1), PcVps75F-K5/(H3–H4)_2_ (molar ratio at 1:1), PcVps75ΔC/(H3–H4)_2_ (molar ratio at 1:1) or PcVps75ΔC-K5/(H3–H4)_2_ (molar mass ratio at 1:1) was added as a substrate. At each reaction interval, SDS-PAGE loading buffer was added to quench the reaction. A control sample (free of HAT) was included to monitor the assay conditions. Western blot analyses using antibodies against H3K9ac and H3K27ac and histone H3 were performed. When necessary, antibodies against H3K56ac were used. The antibodies and dilutions used were anti-H3 (1:1000), anti-H3K9ac (1:1000, Zen bioscience, China), anti-H3K27ac (1:1000, Biolab, USA), and anti-H3K56ac (1:1000).

### Vps75-Rtt109 in vivo binding assay

*S. cerevisiae* *rtt109*Δ *vps75*Δ *gcn5*Δ triple deletion mutants were used along with standard yeast medium and genetic manipulations. The genes *PCVPS75F, PCVPS75F-K5, PCVPS75ΔC, PCVPS75ΔC-K5* and *PCRTT109* were cloned into the expression vector pnTAP416 with a protein A tag and into pHis415 with a GFP tag using the double enzyme digestion method. To test whether PcVps75 binds to PcRtt109 in vivo, plasmids containing either Vps75-pnTAP416, Rtt109-GFP-pHis415, or vectors were transformed into the *rtt109*Δ *vps75*Δ *gcn5*Δ triple deletion strain. The volume of yeast cells was estimated, and an equal volume of IP buffer (25 mm Tris, pH 8.0, 100 mm NaCl, 1 mm EDTA, 10 mm MgCl_2_, 0.01% NP-40, and 1 mm DTT) with protease inhibitors (1 mm PMSF, 1 mm benzamidine, 1 mm aprotinin, 10 mm sodium butyrate) and 15 kU/ml DNase I were used to resuspend yeast cells. The yeast cells were crushed by glass beads beating. The resulting lysate was cleared by centrifugation at 13,000 g for 30 min at 4 °C, and the supernatant was incubated with IgG Sepharose beads or GBP Sepharose beads for 3 hours. Western blot analyses using antibodies against GFP were performed to recognize the GFP-tagged PcVps75 and Protein A tagged PcRtt109 (Protein A can be detected by any antibody). β-actin was used as an internal reference. The antibodies and dilutions used were anti-GFP (1:10,000, Ruiyingbio, China) and anti-β-actin (1:10,000, AB clonal, China). Antibodies against H3K56Ac and H3 were made in the laboratory.^42^

### Detection of the acetylation level of genotoxin sensitivity assays

We expressed PcVps75F, PcVps75F-K5, PcVps75∆C, and PcVps75∆C-K5 with PcRtt109 in yeast using SCM-Leu/Ura medium. Meanwhile, the pnTAP416 vector was added as the blank control. H3K9ac, H3K27ac, and H3K56ac were detected in mutant yeast cells in the presence of PcRtt109 and PcVps75 after 6 hours of induction with 2% galactose at 30 °C. Western blotting was performed to examine the total proteins from yeast cells as shown, with antibodies against H3K9ac, H3K27ac, H3K56ac and histone H3.

We assessed the growth of PcRtt109-complemented yeast in the presence or absence of camptothecin. After the overnight growth of various strains at 30 °C in medium with 2% raffinose, yeast cells were diluted to ~ 6 × 10^6^ cells/ml. Yeast cells were then tenfold serially diluted and plated on medium plates containing 2% galactose in the presence of the DNA-damaging agent camptothecin (1.5 μm). Next, yeasts were cultured for three additional days, and growth was assessed. Images were photographed using a digital camera (Cannon, USA).

### The construction of a phylogenetic tree of Rtt109s in fungi

The evolutionary history of Rtt109s in fungi was inferred using the neighbor-joining method.^43^ The optimal tree with the sum of branch length = 9.23066341 is shown. The percentage of replicate trees in which the associated taxa clustered together in the bootstrap test (500 replicates) are shown next to the branches.^44^ The tree is drawn to scale, with branch lengths in the same units as those of the evolutionary distances used to infer the phylogenetic tree. The evolutionary distances were computed using the p-distance method^45^ and are in units of the number of amino acid differences per site. The analysis involved 41 amino-acid sequences. All positions with <50% site coverage were eliminated. That is, fewer than 50% alignment gaps, missing data, and ambiguous bases were allowed at any position. There were a total of 410 positions in the final dataset. Evolutionary analyses were conducted in MEGA7.^2^

### Protein data bank accession code

The structure factors and atomic coordinates have been deposited in the Protein Data Bank with the PDB ID codes 5YPS and 5ZB5.

## Supplementary information

supplementary data
