# Supplementary material for: Structural basis for the acetylation of histone H3K9 and H3K27 mediated by the histone chaperone Vps75 in Pneumocystis carinii
Source: Signal Transduct Target Ther. 2019 May 10;4:14. doi: 10.1038/s41392-019-0047-8 (PMC6509256; doi:10.1038/s41392-019-0047-8)
Supplement: Supplementary file 1 — supplementary data [file 41392_2019_47_MOESM1_ESM.pdf]

Supplementary data

Figure S1. PcRtt109 cannot interact with PcVps75 in vitro.

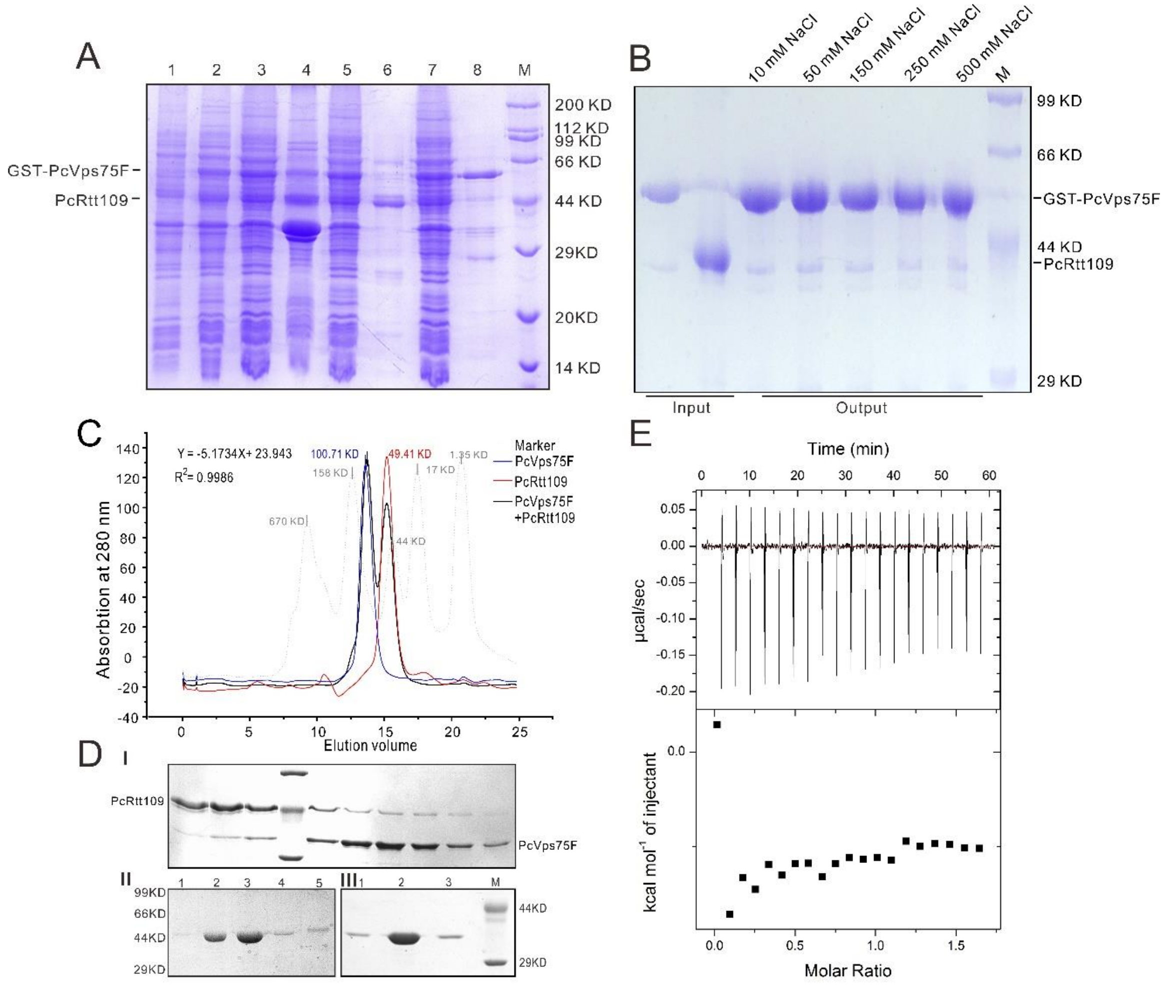

(A) Co-expression of GST-PcVps75F and 6-His-PcRtt109. Lanes 1: the whole-cell lysate of negative controls; Lanes 2: induced whole-cell lysate; Lanes 3–6 are the result for 6-His: Lane 3/4: the supernatant/precipitant of induced whole-bacterial lysate; Lane 5: flow through from Ni-NTA affinity chromatography; Lane 6: the purified 6-His eluted from Ni-NTA affinity chromatography. Lanes 7–8 are the affinity chromatography result for GST-PcVps75F; Lane 7: flow through from GST affinity chromatography; Lane 8: the purified GST-PcVps75F eluted from GST affinity chromatography; “M” represents the protein marker indicated on the right. (B) GST tagged

PcVps75F were incubated with excess amounts of PcRtt109 in different salt concentrations varying from 10 mM to 500 mM and stained by Coomassie. (C) Analytical gel filtration of PcVps75F (blue line), PcRtt109 (red line) and mixture of two proteins (black line). The peak positions of molecular weight standards are indicated in gray dotted lines. The inset represents Regression equation and R square of the molecular weight calibration curve, performed as in Materials and Methods. (D) SDS-PAGE analysis of analytical gel filtration of PcVps75F (III), PcRtt109 (II) and mixture of two proteins (I). (E) Isothermal titration calorimetry of PcRtt109 and PcVps75F interaction.

Figure S2. Sequence alignment of Vps75 homologs from different species.

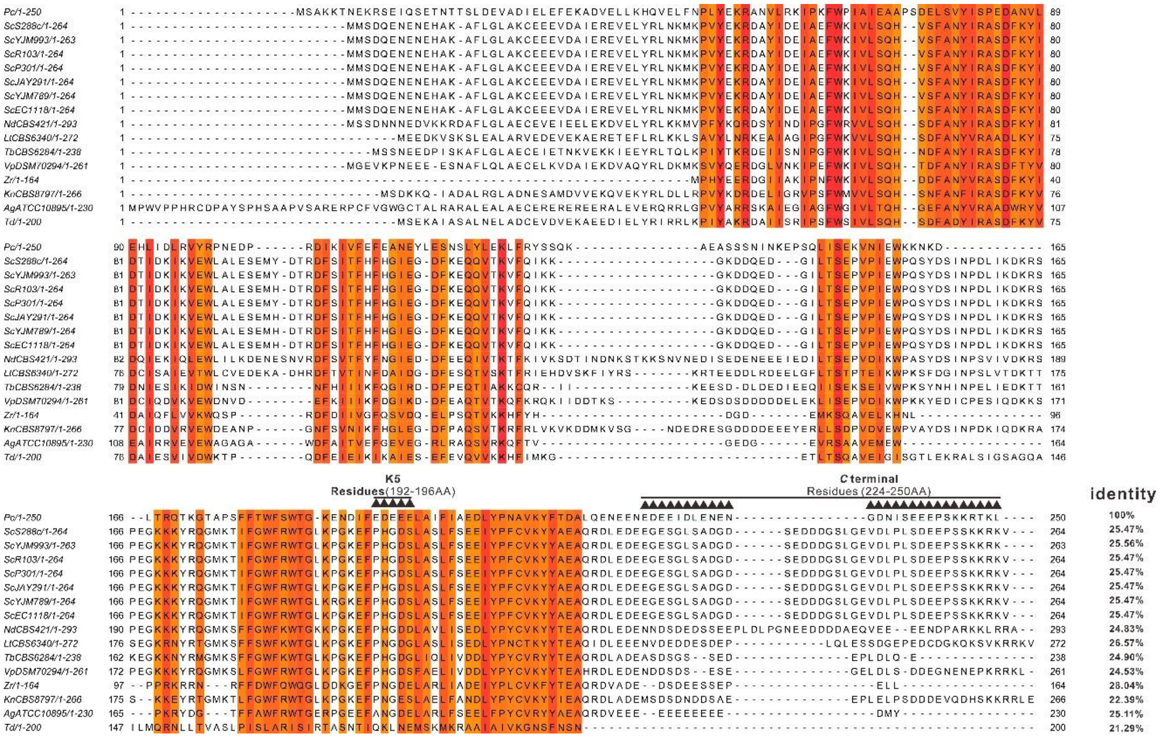

The residue numbers of PcVps75 are at the top indicated as “Pc/1-250”. The identity of PcVps75 to the homologous sequences is shown at right and two main regions for histones interaction are marked with solid triangles.

Figure S3. Comparison of PcVps75ΔC structure with that of SpCcp1, ScVps75, yNAP1, and hSET.

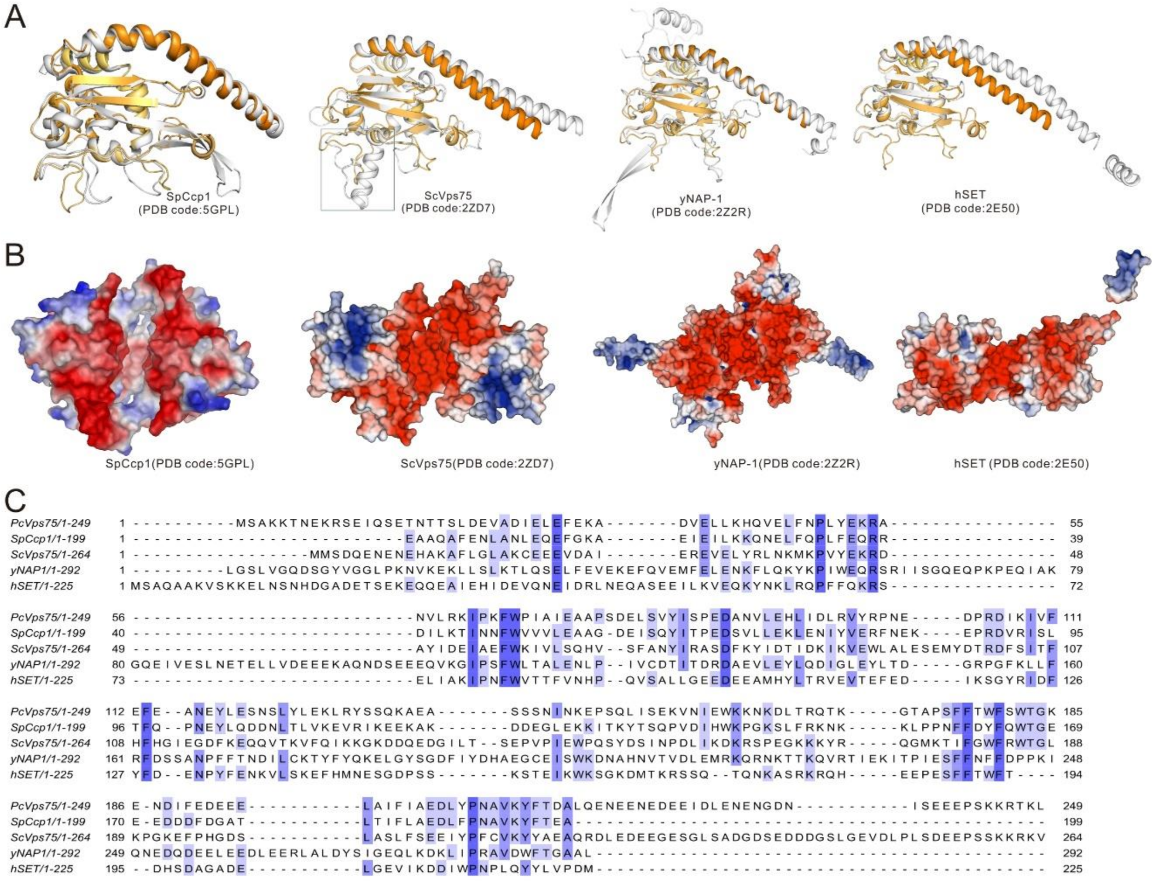

(A) Superposition of PcVps75ΔC monomer and SpCcp1, ScVps75, yNAP1, and hSET. The major difference in the domain II of PcVps75ΔC and ScVps75 is indicated with a black box. (B) Electrostatic potential mapped onto the molecular surface of homolog models of SpCcp1, ScVps75, yNAP1, and hSET. Red and blue colored regions denote negative and positive charges, respectively. (C) The alignment compares PcVps75ΔC, SpCcp1, ScVps75, yNAP1, and hSET. Residues are colored according to percent conservation with darker blue representing higher conservation.

Figure S4. The structural basis for the interaction between Vps75 and Rtt109.

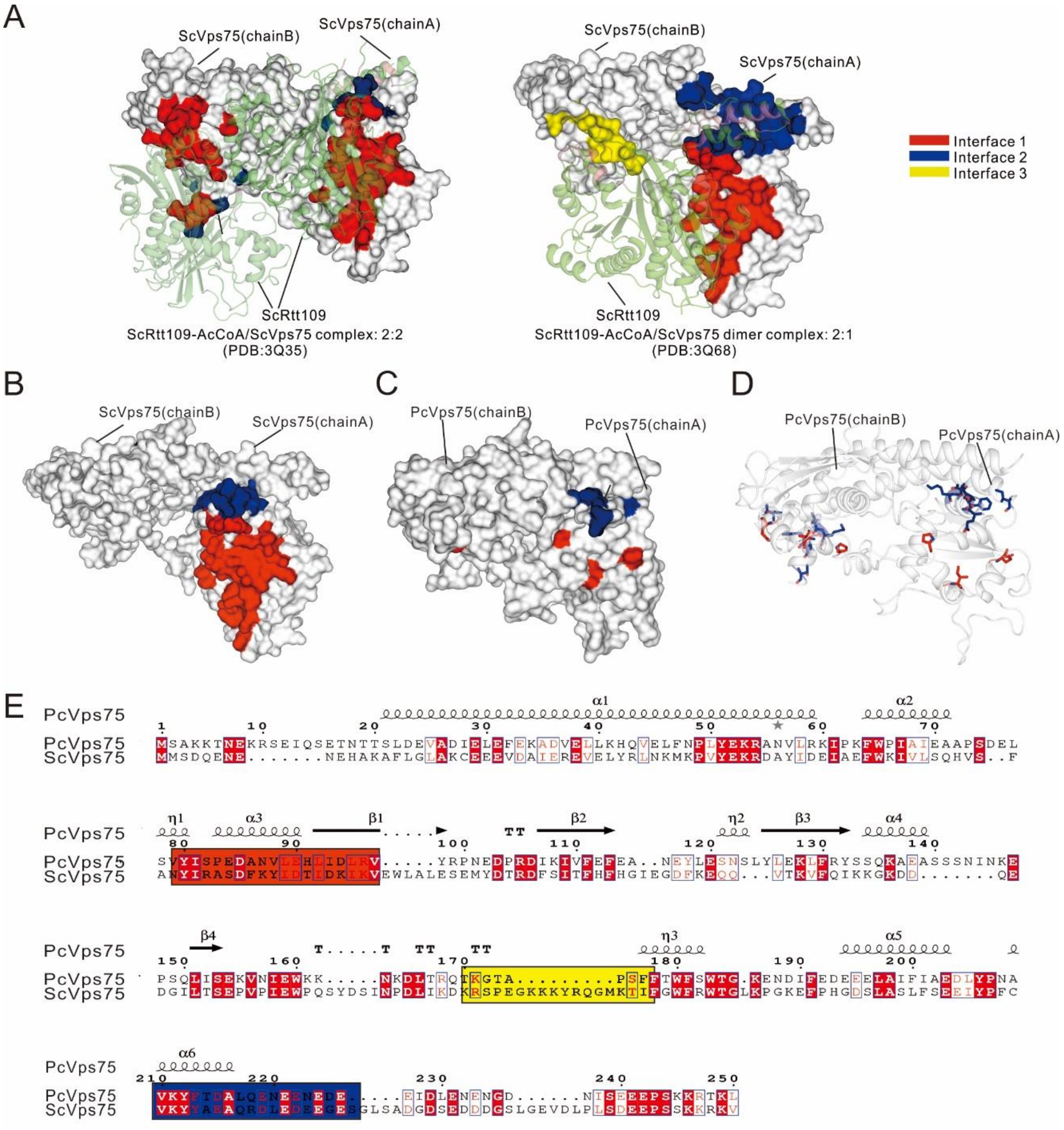

(A, B) Structure comparison of the two stoichiometric ratios of solid complex of ScRt109 and ScVps75. The main interfaces are colored blue, red, and yellow. (B-D) The interfaces in PcVps75 aligned with ScVps75, shown as surface and sticks and colored blue and red. (E) The sequence alignment of PcVps75 and ScVps75. The secondary structure and the interfaces homolog to

ScVps75 are indicated. The interfaces are marked in red, yellow, and blue boxes.

Figure S5. The "loop-α-helix-loop" motif in ScRtt109 is critical for the interaction with Vps75s.

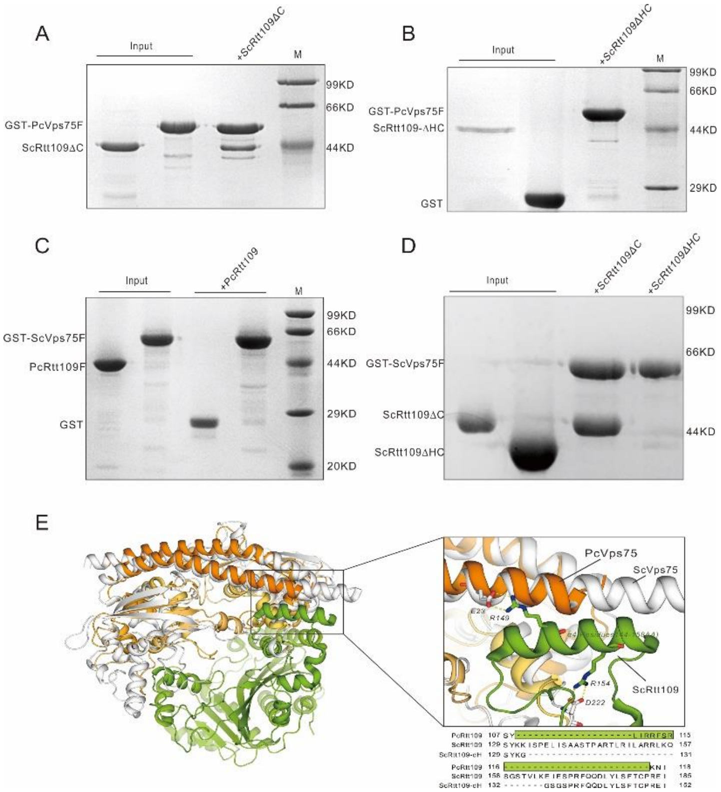

(A, B) GST pulldown assays of interaction between PcVps75F and ScRtt109ΔC or ScRtt109ΔHC. (C) GST pulldown assays of interaction between ScVps75F and PcRtt109. (D) GST pulldown assays of ScVps75F with ScRtt109ΔC and ScRtt109ΔHC. (E) Structure alignment of ScVps75 with PcVps75 and sequence alignment of ScRtt109 with PcRtt109. The main interface in the ScRtt109-ScVps75 complex is indicated in the black box (detail is shown in the right panel). The key interacting residues in ScRtt109 and ScVps75 are shown as sticks and colored green and white, respectively. The key salt bonds are shown as dotted lines. The homolog sequence lost in PcRtt109 is indicated by green boxes.

Figure S6. Sequence alignment of Rtt109s in fungi.

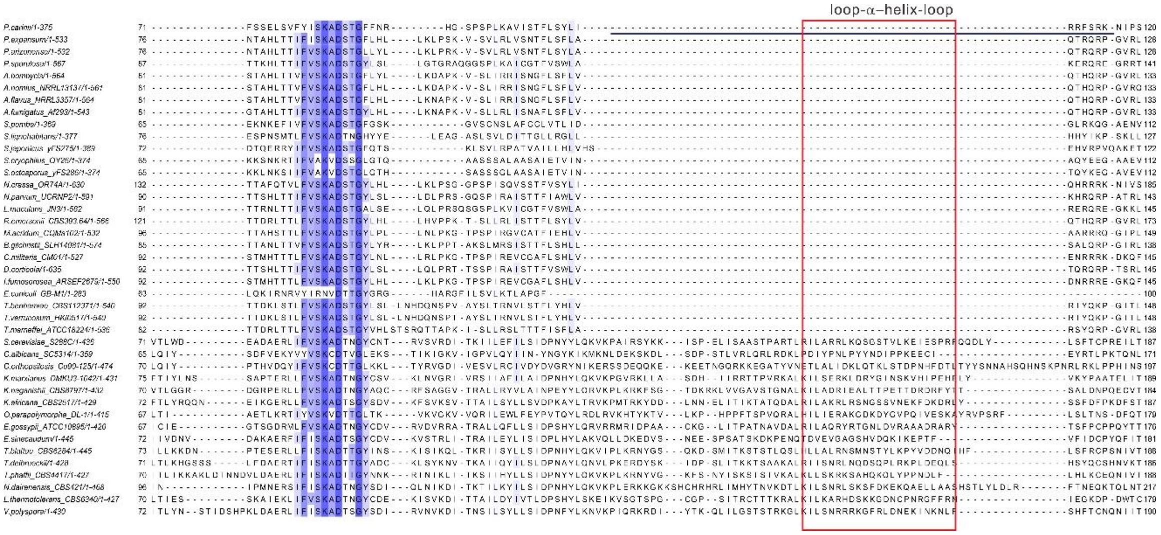

Residues are colored according to percent conservation with darker blue representing higher conservation. PcRtt109 at the top is underlined in blue. The 130-179 segment is indicated in the red box.

Figure S7. The analytical gel-filtration profile of the mixture of PcVps75/PcRtt109/$(H3\text{-}H4)_2$.

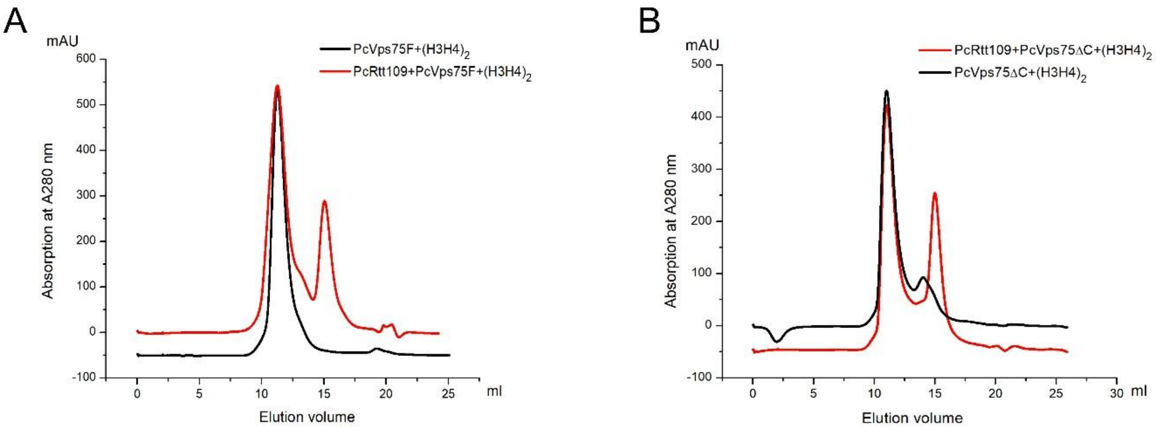

Analytical gel filtration profile of PcVps75F/PcRtt109/$(H3\text{-}H4)_2$ (A) or PcVps75ΔC/PcRtt109/$(H3\text{-}H4)_2$ (B). The mixture of Vps75F-$(H3\text{-}H4)_2$/Vps75ΔC-$(H3\text{-}H4)_2$ is shown as black line and the mixture of three proteins is shown as red line.

Figure S8. Phylogenetic tree of Rtt109s in different fungal species.

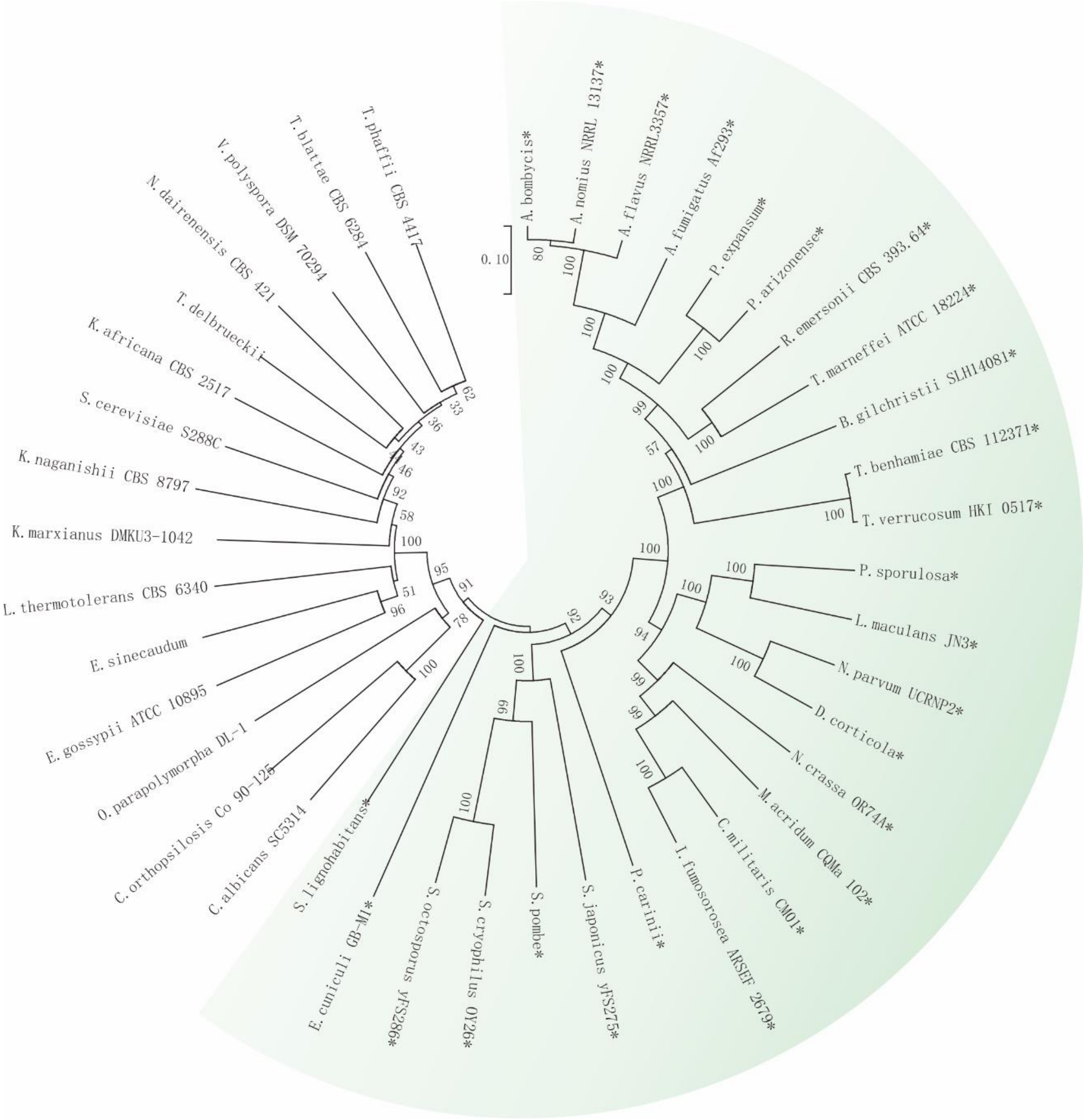

The condensed circular tree of the Rtt109 genes in different fungal species. Rtt109 proteins without the “loop-α-helix-loop” motif are marked with an asterisk and shadowed with green fan.

Figure S9. Two of calcium clusters in the PcVps75 tetramer.

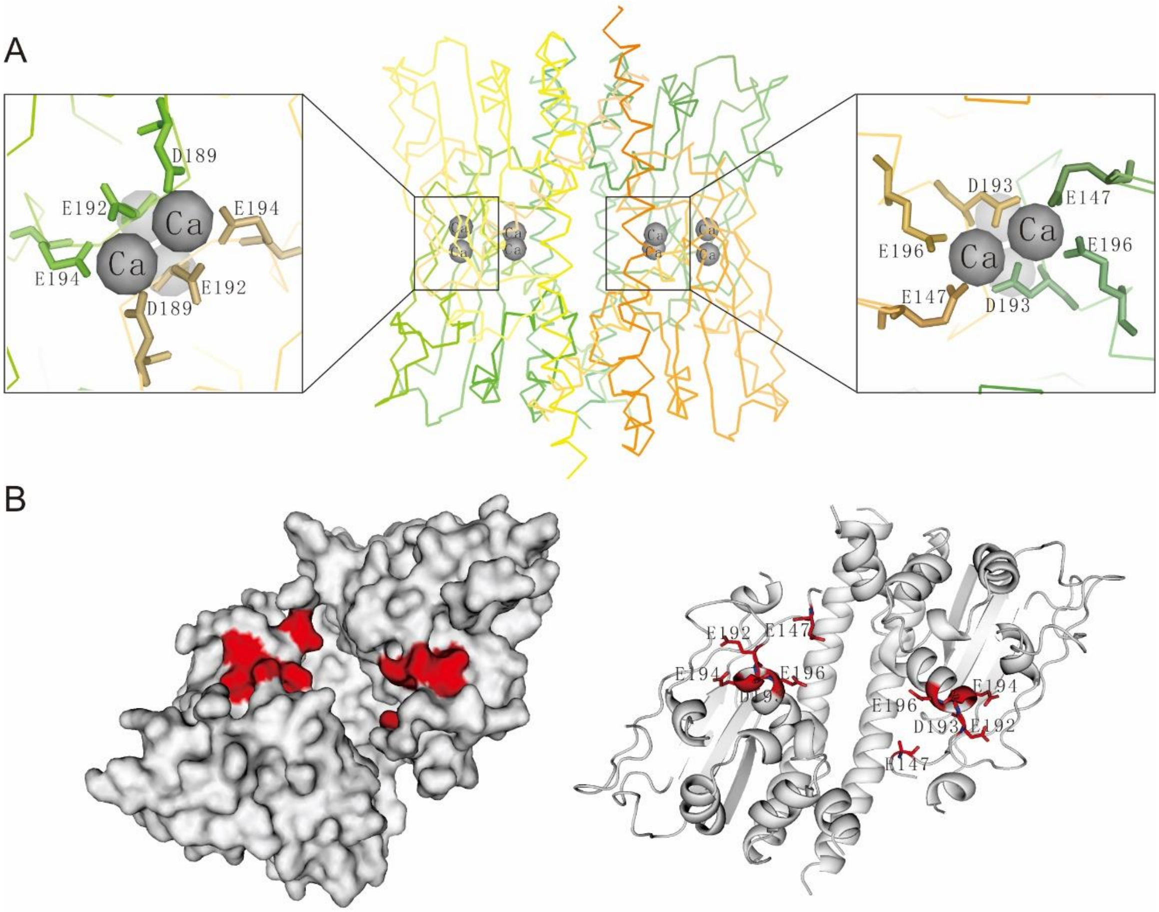

(A) The structure of PcVps75 is shown as ribbon. “Ca” represents calcium ion. The residues in PcVps75 interacting with calcium are shown as sticks. (B) The surface of the protein responsible for calcium ion binding and related residues in calcium ion clusters in PcVps75 are colored red.
